# Lineage Landscape: a comprehensive database that records lineage commitment across species

**DOI:** 10.1093/nar/gkac951

**Published:** 2022-10-28

**Authors:** Haoteng Yan, Ronghao Wang, Shuai Ma, Daoran Huang, Si Wang, Jie Ren, Changfa Lu, Xin Chen, Xiaoyong Lu, Zikai Zheng, Weiqi Zhang, Jing Qu, Yuanchun Zhou, Guang-Hui Liu

**Affiliations:** Advanced Innovation Center for Human Brain Protection, and National Clinical Research Center for Geriatric Disorders, Xuanwu Hospital Capital Medical University, Beijing 100053, China; State Key Laboratory of Membrane Biology, Institute of Zoology, Chinese Academy of Sciences, Beijing 100101, China; State Key Laboratory of Stem Cell and Reproductive Biology, Institute of Zoology, Chinese Academy of Sciences, Beijing 100101, China; Institute for Stem cell and Regeneration, CAS, Beijing 100101, China; Beijing Institute for Stem Cell and Regenerative Medicine, Beijing 100101, China; Aging Translational Medicine Center, International Center for Aging and Cancer, Beijing Municipal Geriatric Medical Research Center, Xuanwu Hospital, Capital Medical University, Beijing 100053, China; Computer Network Information Center, Chinese Academy of Sciences, Beijing 100190, China; State Key Laboratory of Membrane Biology, Institute of Zoology, Chinese Academy of Sciences, Beijing 100101, China; Institute for Stem cell and Regeneration, CAS, Beijing 100101, China; Beijing Institute for Stem Cell and Regenerative Medicine, Beijing 100101, China; Computer Network Information Center, Chinese Academy of Sciences, Beijing 100190, China; Advanced Innovation Center for Human Brain Protection, and National Clinical Research Center for Geriatric Disorders, Xuanwu Hospital Capital Medical University, Beijing 100053, China; Aging Translational Medicine Center, International Center for Aging and Cancer, Beijing Municipal Geriatric Medical Research Center, Xuanwu Hospital, Capital Medical University, Beijing 100053, China; The Fifth People’s Hospital of Chongqing, Chongqing 400062, China; Institute for Stem cell and Regeneration, CAS, Beijing 100101, China; University of Chinese Academy of Sciences, Beijing 100049, China; China National Center for Bioinformation, Beijing 100101, China; CAS Key Laboratory of Genomic and Precision Medicine, Beijing Institute of Genomics, Chinese Academy of Sciences, Beijing 100101, China; Computer Network Information Center, Chinese Academy of Sciences, Beijing 100190, China; Computer Network Information Center, Chinese Academy of Sciences, Beijing 100190, China; University of Chinese Academy of Sciences, Beijing 100049, China; China National Center for Bioinformation, Beijing 100101, China; CAS Key Laboratory of Genomic and Precision Medicine, Beijing Institute of Genomics, Chinese Academy of Sciences, Beijing 100101, China; University of Chinese Academy of Sciences, Beijing 100049, China; China National Center for Bioinformation, Beijing 100101, China; CAS Key Laboratory of Genomic and Precision Medicine, Beijing Institute of Genomics, Chinese Academy of Sciences, Beijing 100101, China; Institute for Stem cell and Regeneration, CAS, Beijing 100101, China; University of Chinese Academy of Sciences, Beijing 100049, China; China National Center for Bioinformation, Beijing 100101, China; CAS Key Laboratory of Genomic and Precision Medicine, Beijing Institute of Genomics, Chinese Academy of Sciences, Beijing 100101, China; State Key Laboratory of Stem Cell and Reproductive Biology, Institute of Zoology, Chinese Academy of Sciences, Beijing 100101, China; Institute for Stem cell and Regeneration, CAS, Beijing 100101, China; Beijing Institute for Stem Cell and Regenerative Medicine, Beijing 100101, China; University of Chinese Academy of Sciences, Beijing 100049, China; Computer Network Information Center, Chinese Academy of Sciences, Beijing 100190, China; Advanced Innovation Center for Human Brain Protection, and National Clinical Research Center for Geriatric Disorders, Xuanwu Hospital Capital Medical University, Beijing 100053, China; State Key Laboratory of Membrane Biology, Institute of Zoology, Chinese Academy of Sciences, Beijing 100101, China; Institute for Stem cell and Regeneration, CAS, Beijing 100101, China; Beijing Institute for Stem Cell and Regenerative Medicine, Beijing 100101, China; Aging Translational Medicine Center, International Center for Aging and Cancer, Beijing Municipal Geriatric Medical Research Center, Xuanwu Hospital, Capital Medical University, Beijing 100053, China; University of Chinese Academy of Sciences, Beijing 100049, China

## Abstract

Commitment to specific cell lineages is critical for mammalian embryonic development. Lineage determination, differentiation, maintenance, and organogenesis result in diverse life forms composed of multiple cell types. To understand the formation and maintenance of living individuals, including human beings, a comprehensive database that integrates multi-omic information underlying lineage differentiation across multiple species is urgently needed. Here, we construct Lineage Landscape, a database that compiles, analyzes and visualizes transcriptomic and epigenomic information related to lineage development in a collection of species. This landscape draws together datasets that capture the ongoing changes in cell lineages from classic model organisms to human beings throughout embryonic, fetal, adult, and aged stages, providing comprehensive, open-access information that is useful to researchers of a broad spectrum of life science disciplines. Lineage Landscape contains single-cell gene expression and bulk transcriptomic, DNA methylation, histone modifications, and chromatin accessibility profiles. Using this database, users can explore genes of interest that exhibit dynamic expression patterns at the transcriptional or epigenetic levels at different stages of lineage development. Lineage Landscape currently includes over 6.6 million cells, 15 million differentially expressed genes and 36 million data entries across 10 species and 34 organs. Lineage Landscape is free to access, browse, search, and download at http://data.iscr.ac.cn/lineage/#/home.

## INTRODUCTION

Multicellular organisms undergo complex transcriptomic and epigenomic changes during embryonic development. These changes underlie lineage determination and differentiation and are important steps throughout embryogenesis, especially stages after gastrulation (1–3). Cells from gastrula germ layers differentiate into multiple lineages, which specifically contribute to organogenesis and organism growth. Aberrant gene expression hinders organogenesis and causes a variety of developmental disorders (4). Interestingly, recent studies have shown that some lineage-specific factors or developmental genes are repurposed in aging and disease conditions. These genes are likely to respond to tissue injury or regeneration (5,6), and functional analyses of these genes demonstrate their potential to play driving roles in aging and aging-related diseases (7–10), which echoes the developmental origins of health and disease (DOHaD) theory to some extent (11,12). Therefore, a comprehensive characterization of lineage differentiation during embryonic development and maintenance of cell lineages thereafter would contribute to a thorough understanding of a broad range of biological processes in developmental and aging biology.

High-throughput omics technologies, including genomics, transcriptomics, single-cell transcriptomics and epigenomics, have provided scientists with multidimensional data at an unprecedented resolution (13–16). Studies of embryonic development during fetal stages in human and model organisms, such as mice, have resulted in a wealth of data (17–19). With the rapid accumulation of such sequencing data, establishing a comprehensive lineage differentiation database along the developmental timeline to store, manage and integrate multi-omics sequencing data has thus become an urgent and unmet need.

Several existing databases related to lineage differentiation include MOCA (1), DevOmics (18), Digital Development (19) and DBTMEE (20) etc. However, the most of previous databases are limited in several aspects. Firstly, several databases are limited to single species or single type of omics data, and exploring the same or homologous genes across species or omics modalities is thus difficult. Secondly, most of these databases focus on the early stages of embryonic development (e.g. gastrulation) due to limited sample accessibility, resulting in a lack of characterization or analysis of differentiation of various lineages during post-gastrulation organogenesis and subsequent life stages. Throughout the entire life cycles, a series of transcriptomic and epigenomic changes are crucial for regulating cell fate determination and cell differentiation (21–25). Therefore, a comprehensive database covering the complete developmental stages, as well as aging, across multiple species and omics, is urgently needed to show the whole landscape of lineage differentiation.

Considering the increasing number of lineage differentiation-related studies from a broad spectrum of developmental stages and species, aggregating such data into a database has become imperative for the field (22–24). To this end, we present Lineage Landscape, a novel lineage differentiation database that allows convenient access to multiple high-throughput omics data collected from different species and developmental stages. Currently, all the Lineage Landscape data are manually collected from the literature and retrieved from existing databases. Overall, our database collects data from over 6.6 million cells, 15 million differentially expressed genes (DEGs) and 36 million data entries across 10 species and 34 organ types. Our database will be continually updated with high-quality lineage differentiation omics data and improved functionalities, which will thus provide a valuable resource for the developmental biology community and life scientists more broadly.

## MATERIALS AND METHODS

### Data collection and processing

To obtain comprehensive datasets of lineage differentiation, we searched the literature using PubMed and manually confirmed that the data were publicly available. We downloaded the sequencing data from the Gene Expression Omnibus (25), ArrayExpress (26) and ENCODE (27). For single-cell sequencing datasets, we downloaded the matrix of gene expression and the cell type annotation information. Each dataset was normalized and processed across differentiation stages using Seurat 4.0.5 (28). The pseudo-temporal analysis was performed by Scanpy 1.8.1 (29). For other datasets, we downloaded either bigwig files or gene quantification files. Gene annotation information from different species was downloaded by the BioMart tool from Ensembl (30).

### Differential expression analysis

Differential expression analysis of single-cell sequencing datasets for each cell type at different stages was calculated using the ‘FindMarkers’ function of the Seurat package (*P*_adj_ < 0.05) (28). Before performing the differential expression analysis, we filtered out cases where cell types at one stage were present at fewer than ten cells in the comparison groups. Differential expression analysis of bulk RNA-seq datasets was calculated with DESeq2 1.32.0 (31) across every two different stages (*P*_adj_ < 0.05 and |log_2_FoldChange| > 0.5).

### Web portal

The Lineage Landscape used SpringBoot web framework v2.5.9, and the front end of the server was developed with Vue 2.6.11 (https://v2.vuejs.org/) and Element UI 2.15.6 (https://element.eleme.io/). Metadata and the analysis results were stored in the MongoDB v4.2.0 database. The interactive visualization diagrams were implemented with the Echarts 5.0.2 (https://echarts.apache.org/), igv.2.12.6 (https://igv.org), D3 7.4.4 (https://d3js.org/) and plotly 2.12.0 (https://plotly.com/).

## DATABASE CONTENT AND USAGE

**Figure 1.**
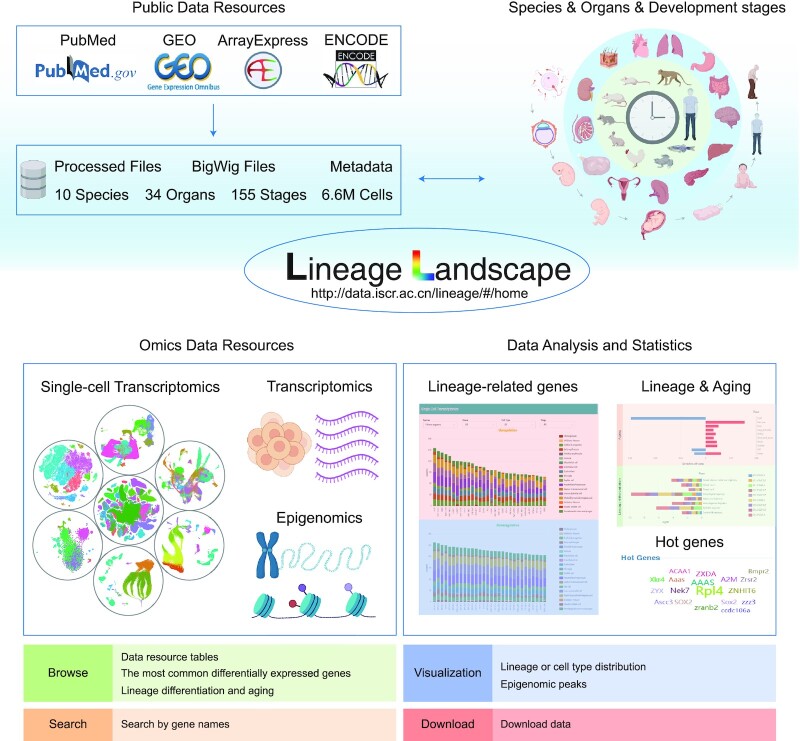
Overview of the Lineage Landscape database. The current implementation of Lineage Landscape includes five modules: Single-cell transcriptomics, Transcriptomics, Epigenomics, Lineage-related genes and Lineage & Aging.

The current implementation of Lineage Landscape (Figure 1) includes three modules, i.e. ‘Single-cell transcriptomics’, ‘Transcriptomics’ and ‘Epigenomics’, to collect omics datasets related to lineage differentiation or development during embryonic, fetal and postnatal stages. In addition, another module ‘Lineage-related genes’ allows users to perform statistical analysis on the results of a large number of DEGs, facilitating the discovery and screening of lineage-specific genes. Finally, the module ‘Lineage & Aging’ helps users to explore the link between lineage development and aging. Overall, our database currently includes over 6.6 million cells, 15 million DEGs and 34 million data entries across 10 species and 34 organs. Lineage Landscape will continue to update and collect high-quality data.

### Single-cell transcriptomics module

The single-cell transcriptomics module collects transcriptomics data during lineage differentiation or development at the single-cell level. It systematically documents cell type-specific changes in gene expression across multiple organs and species, including humans (17,32,33), mice (1,34–36), frogs (22), zebrafishes (23) and so on. This module also provides a collection of high-resolution, comprehensive reference maps of different cell types/subtypes. In this module, users can view the newly published single-cell datasets in the ‘Landscapes’ scroll bar. When users click on an atlas, they will go to the sub-page of the dataset. From that point on, users can easily explore the gene expression patterns of specific cell types, visualize these in an interactive interface, download DEGs list and related information. Within each dataset, users can browse transcriptomic changes for each cell type and compare any two developmental stages. Moreover, as pseudo-temporal analysis is very valuable for the study of lineage development or commitment, the results of pseudo-temporal analysis for some datasets were in-built into our database. In the future, with more robust and less laborious bioinformatics tools, we hope to integrate datasets of millions of cells into pseudo-temporal analysis.

### Transcriptomics module

Bulk level RNA-seq data can be a powerful complement to single-cell sequencing data as it has the advantage of greater sequencing depth, which is important for assessing the overall gene expression changes during development. Hence, we developed a transcriptomics module that currently contains >5.9 million DEGs identified during embryonic, fetal and postnatal stages across seven species. Users can find genes of interest through a convenient keyword search function, and discover genes that are expressed during early development but silenced after maturation. Furthermore, users can discover whether any of these developmental stage-specific genes are reactivated during aging, providing a reference list for potential antagonistic pleiotropic factors. In addition, users can also click on the picture of the corresponding dataset to view and download all DEGs in this article. Over time, we will continue collecting more high-quality datasets, and we encourage users to upload their data to the database. Thus, in this module, we have integrated a large amount of RNA sequencing data from published developmental biology research articles, making it possible to cross-check the expression changes of any gene during embryonic, fetal, and postnatal stages.

### Epigenomics module

This module provides users with a tool to query epigenomic information during embryogenesis or organogenesis across species and organs. Currently, it mainly contains chromatin immunoprecipitation sequencing (ChIP-Seq) data that identify how specific development-related loci are regulated by histone modifications and transcription factors. It also contains whole genome bisulfite sequencing (WGBS) data for DNA methylation status, Assay for transposase-accessible chromatin sequencing (ATAC-Seq), and DNase I hypersensitive sites sequencing (DNase-Seq) data allowing genome-wide profiling of chromatin accessibility. For example, ChIP-seq of the histone modifications H3K4me1, H3K4me2, H3K4me3, H3K9ac, H3K9me3, H3K27ac and H3K27me3 recently revealed the importance of epigenomics during mouse embryogenesis across 11 organs (27). All data included in this module originate from published high-quality datasets and will be updated continuously. Users can easily visualize histone modifications, DNA methylation, and chromatin accessibility tracks at specified genes or chromosome regions with the IGV tool (37), and can also download the metadata of BigWig files for visualization by other epigenome browsers. Users can also enter specific gene coordinate information to view epigenetic modifications at the specific genomic loci. In conclusion, this module aims to provide a systematic platform to support further research on epigenomic regulation during embryonic, fetal, and postnatal stages.

### Lineage-related genes

Based on the DEGs included in the single-cell transcriptomics and transcriptomics modules, the frequency of genes was counted according to related species, organ, or cell types across different lineage differentiation stages. The ‘Lineage-related genes module’ shows the top 30 most common DEGs according to the users’ filter choice on the website in real-time statistics. The upregulated genes and downregulated genes are counted separately. Users can access genes that frequently change in the transcriptomics and single-cell transcriptomics datasets at different stages during lineage commitment. In addition, genes related to human diseases as annotated by KEGG (38) are listed in tables on the website. For example, *AQP2*, a marker of principal cells in the mammalian collecting duct, plays an important role during human kidney organogenesis (39,40). This gene shows the highest frequency during human kidney development in transcriptomics datasets. In addition, *AQP2* is related to congenital nephrogenic diabetes insipidus, a urinary system disease characterized by renal insensitivity to the antidiuretic effect of arginine vasopressin (41). This module provides a platform for scientists to explore these genes, allowing the identification of possible key regulatory factors during development and diseases.

### Lineage & aging

Aging regulation is considered to utilize, at least part of the molecular mechanisms that control lineage determination and differentiation, as well as maintenance of cell identities in organogenesis and maturation during development, as some of which continued throughout the adult life (42–44). Recent studies have shown that some lineage-specific factors or genes during embryogenesis are repurposed in aging, suggesting an intrinsic link between lineage differentiation and aging (11). To explore the link between lineage development and aging, a single-cell transcriptomic atlas of 20 aging organs in the mice was used to benchmark aging (45). Lineage Landscape users can simultaneously trace transcriptional changes of certain genes in the same lineage or cell type during mouse development and aging. For example, the expression level of *Araf* decreases during mouse hepatocyte and endothelial development but increases during aorta aging. The gene *Nfib* is essential for the development of a variety of organ systems (46) and tends to be upregulated during aging in mice, especially in secretory glands or organs. All these results tracing lineage development or aging may inspire further studies. In addition, in order to facilitate browsing of relevant genes in other datasets, users can click the gene name displayed on the leftmost screen to perform a quick full database search for the gene. Users can explore trends of the same genes during development and aging.

## CONCLUDING REMARKS

The current implementation of the Lineage Landscape database has several advantages for broad fields as follows. (i) Lineage Landscape encompasses datasets collected from wide-ranging from fertilization to old age across different species and omics datasets, including transcriptomics, single-cell transcriptomics and epigenomics, and will expand to genomics and proteomics in the future. (ii) Lineage Landscape provides a list of lineage differentiation-related genes and contains search functions for specific gene names across different modules. Lineage Landscape provides users with interactive and user-friendly functionalities that enable the exploration of specific gene expression changes associated with lineage differentiation. (iii) Lineage Landscape collects lineage information from early embryogenesis to postnatal development, even including data from aged individuals, allowing researchers to explore links between lineage development and aging. In addition, based on the existing literature and database, we have recorded the association of genes between lineage differentiation and human diseases. In the future, we will continue to add more high-quality omics data, as well as bioinformatic tools. At the same time, we encourage users to communicate with Lineage Landscape for data dissemination and sharing. Indeed, Lineage Landscape is bound to become an important resource for the broader life sciences community.

## DATA AVAILABILITY

All data in Lineage Landscape is available to researchers (http://data.iscr.ac.cn/lineage/#/home). Users can directly download search results in the corresponding modules without registration or login.
